# Phylogenetic Placement of Exact Amplicon Sequences Improves Associations with Clinical Information

**DOI:** 10.1128/mSystems.00021-18

**Published:** 2018-04-17

**Authors:** Stefan Janssen, Daniel McDonald, Antonio Gonzalez, Jose A. Navas-Molina, Lingjing Jiang, Zhenjiang Zech Xu, Kevin Winker, Deborah M. Kado, Eric Orwoll, Mark Manary, Siavash Mirarab, Rob Knight

**Affiliations:** a Department of Pediatrics, University of California San Diego, La Jolla, California, USA; b Department of Computer Science and Engineering, University of California San Diego, La Jolla, California, USA; c University of Alaska Museum and Department of Biology and Wildlife, Fairbanks, Alaska, USA; d Departments of Family Medicine & Public Health and Medicine, University of California San Diego, La Jolla, California, USA; e Department of Medicine, Bone and Mineral Unit, Oregon Health and Sciences University, Portland, Oregon, USA; f Department of Pediatrics, Washington University, St. Louis, Missouri, USA; g Center for Microbiome Innovation, University of California San Diego, La Jolla, California, USA; h Department of Electrical and Computer Engineering, University of California San Diego, La Jolla, California, USA; Mayo Clinic

**Keywords:** SEPP, amplicon sequencing, microbial community analysis, phylogenetic placement

## Abstract

Recent algorithmic advances in amplicon-based microbiome studies enable the inference of exact amplicon sequence fragments. These new methods enable the investigation of sub-operational taxonomic units (sOTU) by removing erroneous sequences. However, short (e.g., 150-nucleotide [nt]) DNA sequence fragments do not contain sufficient phylogenetic signal to reproduce a reasonable tree, introducing a barrier in the utilization of critical phylogenetically aware metrics such as Faith’s PD or UniFrac. Although fragment insertion methods do exist, those methods have not been tested for sOTUs from high-throughput amplicon studies in insertions against a broad reference phylogeny. We benchmarked the SATé-enabled phylogenetic placement (SEPP) technique explicitly against 16S V4 sequence fragments and showed that it outperforms the conceptually problematic but often-used practice of reconstructing *de novo* phylogenies. In addition, we provide a BSD-licensed QIIME2 plugin (https://github.com/biocore/q2-fragment-insertion) for SEPP and integration into the microbial study management platform QIITA.

**IMPORTANCE** The move from OTU-based to sOTU-based analysis, while providing additional resolution, also introduces computational challenges. We demonstrate that one popular method of dealing with sOTUs (building a *de novo* tree from the short sequences) can provide incorrect results in human gut metagenomic studies and show that phylogenetic placement of the new sequences with SEPP resolves this problem while also yielding other benefits over existing methods.

## Abstract (precis)

The move from OTU-based to sOTU-based analysis, while providing additional resolution, also introduces computational challenges. We demonstrate that one popular method of dealing with sOTUs (building a *de novo* tree from the short sequences) can provide incorrect results in human gut metagenomic studies and show that phylogenetic placement of the new sequences with SEPP resolves this problem while also yielding other benefits over existing methods.

## INTRODUCTION

**FIG 1:**
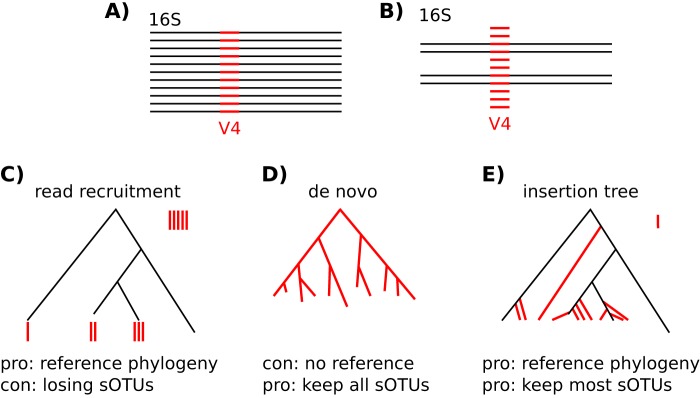
Comparing read recruitment, *de novo*, and insertion tree strategies for phylogenetic diversity computation. (A) Ideally, all short amplicon fragments (red) would have known full-length 16S sequences (black), which in turn would allow reconstruction of a phylogenetic tree. (B) In real-world experiments, only a minority of fragments have corresponding full-length 16S references. (C) The “read recruitment” strategy, also known as closed-reference OTU picking, assigns fragments to tips of a well-curated reference phylogeny, e.g., Greengenes, with a given sequence similarity threshold. Fragments of clades not covered in the reference are rejected. (D) In order to keep all fragments, the *de novo* strategy reconstructs the whole phylogeny based on the short fragments that do not carry as much evolutionary signal as full-length 16S sequences and thus often results in topologically very different trees. (E) The insertion tree strategy takes advantage of a well-curated phylogeny and extends it with fragments obtained by experiment. Only highly unrelated fragments are rejected, while the overall topology of the resulting phylogenetic trees remains stable.

Recent algorithmic advances in amplicon-based microbiome studies have enabled the derivation of exact amplicon sequence fragments. Instead of the coarse operational taxonomic units (OTUs) that have dominated the field for over a decade, these new methods (e.g., Deblur [1] and DADA2 [2]) enable the investigation of sub-OTUs (sOTUs) through the removal of erroneous sequences and add the ability to analyze amplicon data at maximal resolution. However, as with all short sequencing fragments, they lack sufficient phylogenetic signal to reproduce a reasonable tree (3, 4), introducing a barrier to the use of phylogenetically aware metrics such as Faith’s PD (5) and UniFrac (6), which are used in many studies. At present, researchers often reconstruct a *de novo* phylogeny or perform a read recruitment strategy against an existing reference tree; we illustrate these methods as well as fragment insertion in Fig. 1. The latter approach works well for OTUs but is hindered for sOTUs by the absence of some taxa in the reference database. Although sequence fragment insertion methods such as EPA (7) and pplacer (8) exist, these methods have not been tested with sOTUs from high-throughput amplicon studies using insertion against a broad reference phylogeny. A recent advance was made with SATé-enabled phylogenetic placement (SEPP) (9), which inserts fragment sequences into a large phylogeny using a divide-and-conquer approach, utilizing HMMER (10) to identify putative subtrees followed by pplacer for the actual fragment placement. We benchmarked SEPP using 16S V4 sequence fragments and showed that it outperforms the present “state-of-the-art” approach of reconstructing *de novo* phylogenies and that it provides the necessary addition in resolution to statistically detect significant sample separation along clinical variables. We chose SEPP among the available phylogenetic insertion pipelines because of its scalable divide-and-conquer algorithm. However, to enable fragment insertion into very large 16S reference trees with hundreds of thousands of tips, we had to make several improvements to the SEPP software program, especially in terms of its memory usage. In addition, we provide a BSD-licensed QIIME2 (11) plugin that both Apple and Linux users can readily integrate into their analyses. We have, furthermore, integrated SEPP into QIITA (described in an unpublished paper), a platform that currently manages ~2,000 microbial studies, with centralized storage that holds ~36 million placements for sOTU fragments of different lengths and regions. New studies and meta-analyses will be processed significantly faster as placements for the majority of affected sOTUs are already available in this common resource.

## RESULTS

### *De novo* phylogenies.

**FIG 2:**
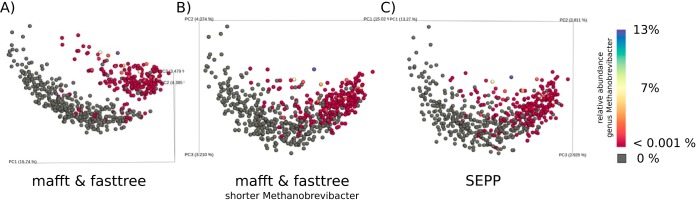
SEPP avoids artificially long outgroup branches that would lead to exaggerated separation in beta diversity data. (A) Principal-coordinate analysis (PCoA) of unweighted UniFrac distances based on a *de novo* phylogeny. Three low-abundance *Methanobrevibacter* sOTUs, not detectable in the lower gray cluster and of very low abundance in the upper colored cluster, drove a spurious separation of 599 stool samples obtained from participants of the MrOS Study. (B) Manually shortening the grandparent’s branch length from 0.82 to 0.4 in the *de novo* phylogeny reunited spurious clusters. (C) Inserting *de novo* fragments into a well-curated reference phylogeny via SEPP also resolved cluster separation but did not require any manual manipulation.

We identified a direct risk to biological interpretation with the use of *de novo* phylogenies in analyzing a 16S microbiome data set composed of human fecal samples collected from 599 men aged 78 to 98 years in the Osteoporotic Fractures in Men (MrOS) Study (12). In that study, a *de novo* phylogeny was constructed from Deblur sOTUs (via QIIME2's Deblur plugin with default parameters) following the steps illustrated in the QIIME 2 Moving Pictures tutorial version 2017.12, i.e., using multiple-sequence alignment via MAFFT (13) and phylogenetic reconstruction via FastTree (QIIME2 uses a FastTree version with double precision) (14). Principal-coordinate analysis (PCoA) of unweighted UniFrac distances showed major differences among samples that could not be explained by clinical information. The only identifiable factor was the presence of a single archaeon (genus *Methanobrevibacter*) that was composed of just three low-abundance sOTUs (see Fig. 2A). An assessment of the phylogeny showed a long (1.43) branch in the archaeal clade which was greater in length than the mean tip-to-root distance (0.94). Manually reducing this branch length removed the clustering (see Fig. 2B), suggesting that the tree was introducing artifactual clustering. This idea was reinforced by the fact that this type of bimodal clustering had not been seen in other human fecal studies using OTU-based methods or shotgun metagenomics. We then inserted the sOTU sequences into the 99% Greengenes reference tree (15) using SEPP and observed that the artifactual clustering had indeed been removed (see Fig. 2C). Artificial cluster separation cannot be observed via the application of the weighted UniFrac method to either approach, because *Methanobrevibacter* is of low abundance, or via the use of Bray-Curtis data, because that metric does not operate on a phylogeny and also takes abundance into account, or via the use of Jaccard data, which represents a phylogeny-free and abundance-free metric.

Beta diversity was computed for all 599 samples on the Deblur table; the sample data were rarefied to 5,870 sequences per sample with 4,727 sOTUs (249 nucleotide [nt]) in total as the unweighted UniFrac distance for the three phylogenetic trees.

Since *de novo* tree construction critically depends on the multiple-alignment algorithm, we also ran the same analysis as that described for Fig. 2A but with PyNAST (16) instead of MAFFT. PyNAST can align input sequences against a given template alignment to leverage prior knowledge. We used the default 85% Greengenes 13.8 alignment as the template here. The resulting PCoA of unweighted Unifrac distances along the PyNAST-plus-FastTree-generated *de novo* phylogeny comprises the same artifactual clustering (data not shown).

### SEPP phylogenies expose relevant ecological signals.

The higher taxonomic resolution of sub-OTU methods, together with more-precise phylogenetic reconstruction techniques such as SEPP, can be leveraged by phylogenetic distance metrics to expose relevant ecological differences from the results obtained by traditional closed- or open- reference OTU picking. We exemplify this potential with two independent real-world microbial studies as follows.

### (i) Malawi children.

**FIG 3:**
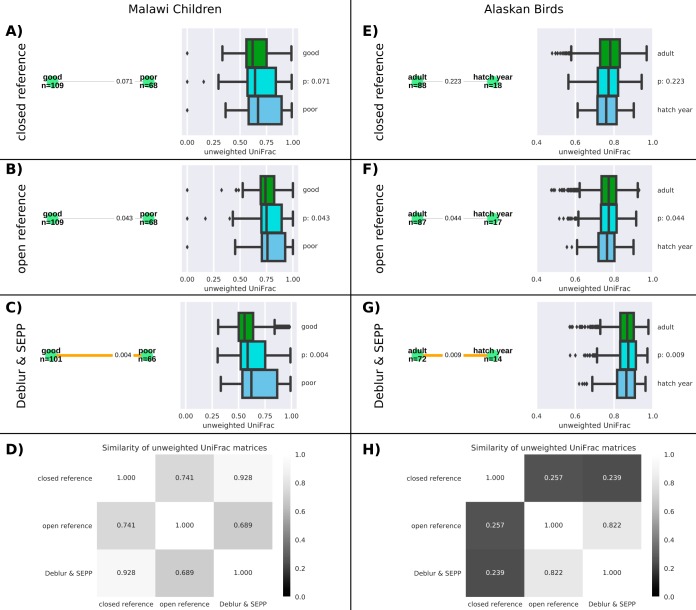
Higher sub-OTU resolution, in combination with SEPP phylogenies, exposed relevant ecological signals. (A to D) For the Malawi children, the same 7,554,708 reads from 179 samples (150 nt; mean number of reads per sample, 42,205) were processed by “closed-reference” OTU picking (A), “open-reference” OTU picking against the same reference database (B), and the sub-OTU method “Deblur” (C), and correlation via Mantel tests for unweighted Unifrac beta diversities were computed (D). (E to G) For the Alaska birds, a total of 5,932,450 reads from 137 samples (125 nt; mean number of reads per sample, 43,303) were processed with both methods mentioned above. Pairwise testing between sample groups was performed via PERMANOVA with 9,999 permutations. Statistically significant differences between groups are indicated via bold orange edges, while nonsignificant edges are colored gray. Green boxes at the right side of panels A, B, and C summarize pairwise beta diversity distances within the group of “good” samples, and the dark blue boxes represent distances within “poor” samples. The cyan-colored boxes show between-group distances, i.e., all pairwise distances between “good” and “poor” samples. Similarly, the green, dark blue, and cyan boxes in panels E (closed-reference OTU picking), F (open-reference OTU picking), and G (Deblur) summarize pairwise distances within “adult” and “hatch year” data and between samples, respectively, and correlation via Mantel tests for unweighted Unifrac beta diversities were computed (H).

Fecal samples from 179 children in a food intervention study (17) were collected from children who were 11.3 (± 0.8) months of age. Child growth was determined as Δ_HAZ_, where Δ_HAZ_ represents the difference between the “height for age *z*-score” at sample collection and that recorded at enrollment (6 months). The data were classified into the categories of “poor” (Δ_HAZ_ value of less than −0.75) and “good” (Δ_HAZ_ value of more than −0.25) growers. Sampled children were chronically undernourished and generally had bad gut health (assessed via mannitol-lactulose tests). The same demultiplexed raw reads were rarefied to 11,000 reads per sample for “closed-reference” picking via QIIME1’s script parallel_pick_otus_sortmerna.py with default parameters and were rarefied to 12,500 reads per sample for “open-reference” picking (using QIIME1’s script pick_open_reference_otus.py with default settings) and to 7,500 reads per sample for Deblur. Different rarefaction depths were required due to very different quality control levels; e.g., Deblur typically filters out ~50% of sequences. Beta diversity was computed in terms of unweighted UniFrac data for all three resulting feature tables along the reference phylogeny of Greengenes 13.8 (97% for closed-reference data), with a reconstructed phylogeny for open-reference data (using QIIME1’s default: aligning short fragments into a ribosomal full-length reference alignment and building a tree via FastTree) and an insertion tree constructed by using SEPP for the feature table produced by the use of Deblur (“Deblur and SEPP”). The correlation (assessed via Mantel tests) between the beta distance matrices of Deblur and SEPP and the closed-reference data was high at 0.93 (*P* < 0.01) (see Fig. 3H). However, the gain in resolution renders the results of a permutational multivariate analysis of variance (PERMANOVA) (18) test with 9,999 permutations between “good” and “poor” growers statistically significant, assuming a significance level of 0.01, for Deblur and SEPP (Fig. 3G), while the results of same test performed on closed-reference data are not (Fig. 3E). The Greengenes reference is engineered for analyses of human gut microbiota; thus, the lower correlation between the open-reference data and the other two methods might indicate inaccuracies of the reconstructed phylogeny. Despite these imprecisions, the significance value of the PERMANOVA test data (see Fig. 3F) was improved compared to the closed-reference data but cannot meet the required threshold of 0.01. In this example, only the combination of Deblur and SEPP can statistically reliably detect differences in the gut microbial composition of children showing “good” versus “poor” growth.

### (ii) Alaskan birds.

Fecal samples or fecal material from the gut was sampled from nine codistributed bird species that breed on the Alaska mainland and throughout the Aleutian Islands. The sampled individuals (all male) were in two different developmental stages: hatch year and adult. Identically to the experiment described above, we processed the same reads with three different methods. Samples were rarefied to 2,000 reads for the open-reference analyses and Deblur and SEPP analyses and to 1,000 reads for the closed-reference analyses. Pairwise testing between sample groups was performed via PERMANOVA (18) with 9,999 permutations. As described above, only the higher taxonomic resolution and precision of the phylogeny of Deblur and SEPP enabled detection of significant differences between “hatch year” and “adult” (*P* < 0.01) (compare the data corresponding to the bold orange edge in Fig. 3C). This finding is in line with multiple other observations of aging or developing gut microbiota (19). Notably, the correlation of beta distances revealed by comparisons of the Deblur and SEPP data to the closed-reference data was very low at 0.27 (see Fig. 3D), indicating major gaps in the Greengenes reference collection with regard to bird-derived microbiota and stressing the importance of reference-independent tools such as Deblur and SEPP.

### SEPP better reconstructs phylogenies.

**FIG 4:**
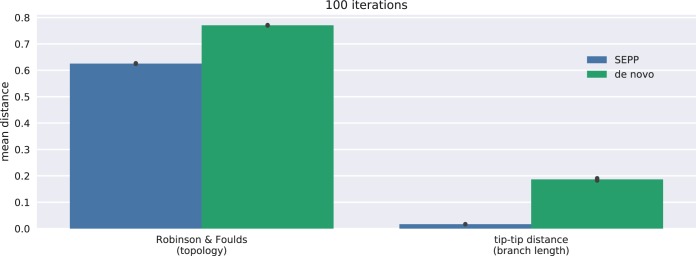
Deviations between *de novo* or insertion trees and gold standard trees. For 100 iterations, we randomly chose 10,000 150-nt V4 fragments to split the Greengenes tree into training and testing trees. Phylogenies for the 10,000 fragments were constructed via QIIME2’s *de novo* recommendations and SEPP. For both metrics, the insertion trees were significantly (two-sided Mann-Whitney tests; *P* < 10^−32^) closer to the gold standard than the *de novo* trees. The tip-to-tip distance summarizes the similarity of two trees as the Pearson correlation coefficient of two sets of path lengths, where pairs with tips not present in both trees are omitted. Those two sets are independently enumerated as pairwise tip-to-tip path lengths for each tree.

In general, methods such as UniFrac are tolerant of noisy phylogenies (20, 21). Nevertheless, improved topology and branch lengths can both improve UniFrac and enhance discriminatory power in comparisons between sample groups; thus, we sought to characterize whether SEPP better recapitulated the reference tree than did the *de novo* approach. To do so, we randomly chose 10,000 (~5%) of all 150-nt V4 fragments (see Materials and Methods) generated from Greengenes 13.8 and removed the corresponding full-length sequences and tips/branches from the 99% reference alignment and the reference tree, respectively. We then reconstructed a *de novo* phylogeny via MAFFT and FastTree for the 10,000 fragments and, in parallel, reinserted the fragments into the reduced Greengenes tree using SEPP. The *de novo* and insertion trees were then compared using tip-to-tip distances (i.e., all pairwise distances between the tips represented by the 5% removed) to a Greengenes tree stripped to the tips of the 10,000 fragments (for ambiguous fragments, one tip was arbitrarily selected), with the insertion tree resulting in a significantly shorter (100 iterations, *P* < 10^−32^ [two-sided Mann-Whitney test]) distance to the stripped Greengenes tree (see Fig. 4). The insertion trees were significantly closer to the stripped trees not only in the comparisons of branch lengths but also in comparisons to data determined by the use of the more coarse-grained metrics that only consider topological features, i.e., Robinson-Foulds distance (22). The methods used in construction of the various trees are described in Materials and Methods.

### Fragment reinsertion.

To test whether SEPP placed sequences correctly in the tree, we then generated V4 fragments for all 1,262,986 Greengenes 13.8 sequences (minus those 1,486 sequences that could not be aligned by PyNAST) and reinserted them into the reference tree without removing tips from the reference tree. Many (87%) of these fragments were unambiguous, i.e., they mapped in a one-to-one manner to a tip in the 99% Greengenes phylogeny based on 203,452 representative tips. However, some fragments were not unique and were able to be derived from multiple tips—we denote these as ambiguous fragments.

**FIG 5:**
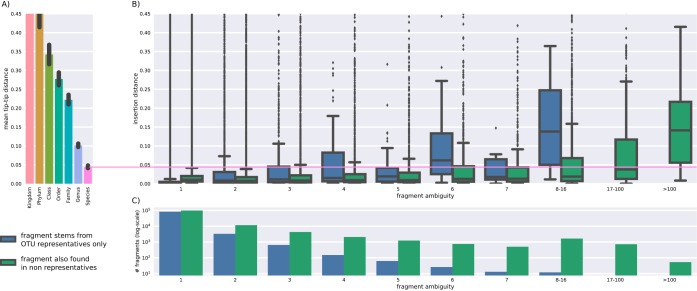
Perfectly matching fragments are precisely inserted below the species level. We extracted all possible (*n* = 208,255) unique V4 150-nt fragments from Greengenes reference alignments and reinserted those into the Greengenes 99% sequence identity reference phylogenetic tree, which is based on 1,261,500 full-length ribosomal sequences. Due to trimming, many full-length sequences map to the same fragment. (A) Taxonomic diversity by rank to establish reference coordinates. (B) Insertion error for V4 fragments as the path length from the inserted position in the tree to the lowest common ancestor (lca) of all true OTU tips. *x*-axis data denote ambiguity, i.e., the number of originating OTUs for a fragment; note the binning for more than 7 true OTUs. Blue bars indicate fragments that map only to representative sequences, while green bars show results for fragments that also map to the majority of nonrepresentative sequences. (C) A histogram for fragment distribution by ambiguity and representativeness.

SEPP employs an ensemble of hidden Markov models (HMM) trained on the alignments associated with subsets of the reference tree to determine if a query sequence should be placed within that specific subset. The reference tree and alignment in our case were built from the representative sequences of the Greengenes 13.8 reference 99% OTUs and included 203,452 tips. Among all 208,255 of our V4 fragments, ~42% stem exclusively from one or more 99% OTU representative sequences (blue bars in Fig. 3 and 5). A single sequence is chosen to represent an OTU (i) if it stems from a named isolate, (ii) if it was a representative in previous releases, or (iii) by sequence length. We therefore assume that insertion of such sequences represents an easier task than insertion of fragments that originate from sequences that are not directly represented in the reference (green bars).

Error was measured by the sum of the branch length to a correct placement for an unambiguous fragment, or the lowest common ancestor of an ambiguous fragment. Unambiguous fragments nearly always fell in the correct placement location (85% within 0.037 branch length), but ambiguous fragments yielded increased errors with increased ambiguity (see Fig. 5). In general, fragments with five or fewer ambiguities were placed close to their lowest common ancestor and were placed below the species level, defined here as a tip-to-tip distance below 0.045.

Rank levels were obtained from SEPP’s reference tree by measuring the maximal tip-to-tip distances within every clade, e.g., within genus *Escherichia*, and by averaging over those distance.

**FIG 6:**
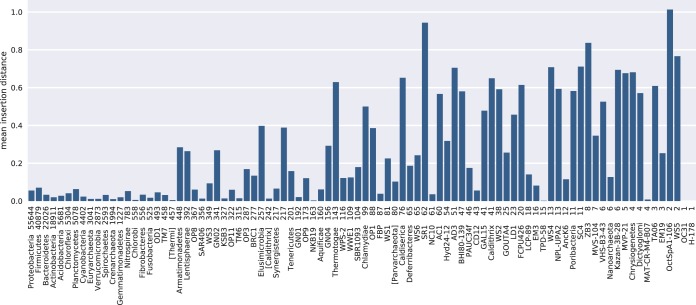
Insertion errors are not equally distributed across the reference phylogeny. *y*-axis data show the mean insertion distance for unambiguous 150-nt V4 fragments grouped by phylum of the true OTUs. Numbers of taxa within phyla are indicated as numbers following phylum names.

Not all phyla tolerated fragment insertion equally, with candidate phyla tending to have poorer performance for unambiguous fragment placement (Fig. 6). This result could have been related to variations in taxon sampling densities among phyla, but no correlations were found between error distribution and phylum size, diversity, or candidate status (data not shown).

### Placement errors grew with fragment deviation.

**FIG 7:**
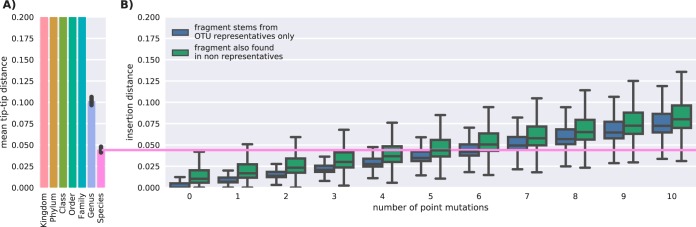
Insertion distance grows linearly with the number of point mutations. (A) Taxonomic diversity reference data were determined as described for Fig. 5. (B) Insertion errors as the path length from insertion to single true OTU node for fragments with up to 10 point mutations.

Next, we simulated novel sequences at defined sequence identities by mutating the unambiguous fragments 1 to 10 times randomly, taking care not to mutate the same position twice and ensuring that the mutated sequences were not already contained in our set. The fragments were then reinserted, and the distance from the insertion point to the OTU tip of the original sequence was measured. As expected, we observed a linear increase in placement distances as a function of the number of mutations introduced (Fig. 7). Assuming an average error rate of 1% for Illumina reads, we expected two read errors per fragment. Despite those two errors, fragments were still precisely inserted below the species level of resolution.

### Open-reference strategy.

Insertion of Deblur fragments into a reference phylogeny via SEPP implicitly follows the open reference (23) strategy. Exact fragments derived from sequences contained in the reference get inserted at positions that were close, i.e., with small branch length, to the corresponding tips. Novel fragments do not need to be discarded, as in closed-reference approaches, but can be inserted with a greater branch length at the best matching subtree. The use of exact sequences instead of OTU identifiers as proxies for taxonomic entities comes with the appealing advantage that the resulting insertion placements are stable across microbiome studies, thus enabling performing of meta-analyses with the same reference phylogeny.

**FIG 8:**
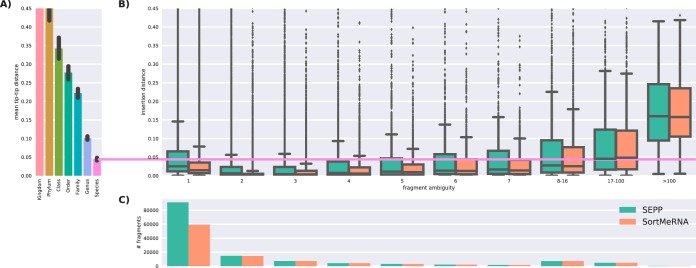
Comparison of insertion errors made by SEPP and SortMeRNA. The reference alignment and tree were randomly split into 10% testing and 90% training sequences. V4 fragments (150 nt) were generated from the test sequences and reinserted via SEPP or aligned via SortMeRNA. (A) Taxonomic diversity by rank to establish reference coordinates. (B) Insertion errors for SEPP and SortMeRNA between the true and assigned positions in the tree. (C) A histogram for fragment distribution by method. Note that SortMeRNA rejected more fragments than SEPP.

To benchmark the ability to handle novel fragments, we again split the Greengenes 13.8 reference into training and testing sets, this time doubling the fraction of removed information to 10% to account for more distantly related taxa. We show in Fig. 8 that the insertion errors made by SEPP were still within the species level and were therefore acceptable. We contrast this performance with that of SortMeRNA (24), a purely sequence-based representative of the closed-reference approach. The insertion error of SortMeRNA is significantly lower up to a fragment ambiguity level of 16 originating OTUs, but that improvement comes with the disadvantage of losing 35% of the unambiguous fragments (compare the leftmost boxes in Fig. 8C) and with the fact that SEPP always inserts fragments with a nonzero branch length even when inserting into the branch that leads to the correct reference sequence.

Most available microbial databases are biased toward human environments, and Greengenes is no exception. Thus, the ratio of lost fragments is likely to grow rapidly for examined environments that differ from the database focus. Open-reference approaches are essential for analyzing such samples.

### Enabling meta-analyses.

*De novo* phylogenies cannot handle amplicons from multiple variable regions, hindering the reuse and integration of these types of data in meta-analyses.

We showcase this by incorporating samples from two independent studies. The "Family" study (25) comprised 854 human samples and 217 dog samples of three body products from a westernized population. The first 128 nt of the V2 region were targeted and sequenced on an Illumina GAIIx system. Running Deblur on the reads trimmed to 100 nt results in 37,181 sOTUs. The “Yanomami” study (26) contained 66 human samples of the same three body products from uncontacted Amerindians in Venezuela. Here, 150-nt V4 fragments were sequenced on a MiSeq platform and processed via Deblur, returning 17,249 sOTUs. The three technical parameters variable region, sequence length, and sequencing platform differed between the studies; those differences might obstruct analyses of biological differences between those samples.

**FIG 9:**
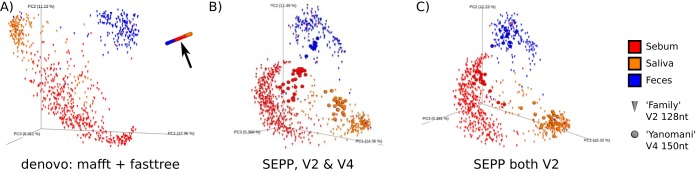
Meta-analyses of two microbiome studies with heterogeneous variable 16S regions. (A) *De novo* tree construction resulted in strong artifacts in the PCoA space (see black arrow). (B) Insertion of heterogeneous sOTUs into the same backbone tree via SEPP resolved the artifact and enabled meaningful insights. (C) Available V2 reads from the “Yanomami” samples served as a positive control. Separation of samples from the two studies was indeed driven by body product and not by different sequencing parameters.

*De novo* construction of a phylogenetic tree for all sOTUs combined from the two studies and subsequent beta diversity computation via unweighted UniFrac analysis of the data in the table listing the nonrarefied combined counts led to the appearance of an obvious artifact in the PCoA space (black arrow in Fig. 9A), where all sample data from the Yanomami study appear in a straight line.

Insertion of the heterogeneous sOTU sequences from the two studies into the same backbone tree via SEPP resulted in a phylogeny that separated the samples as expected in the PCoA space corresponding to the body product data, where the differences between studies are small compared to differences among body sites (Fig. 9B).

Fortunately, the same 66 samples from the Yanomami study were also profiled targeting the V2 region on an Illumina GAIIx system. We could therefore control for all three technical parameters by trimming reads to 100 nt and subjecting them to Deblur, resulting in 6,604 sOTUs. As a positive control, we computed data corresponding to unweighted UniFrac beta diversity between samples from the two studies based on a SEPP-derived phylogeny purely consisting of 100-nt V2 sOTUs. As shown in Fig. 9C, separation was indeed driven by body product and not by study, i.e., not by technical parameters, as one might have wrongly assumed on the basis of the *de novo* results.

### Availability.

The divide-and-conquer approach of SEPP along the reference tree opens up a huge potential for parallelization. Because the placement of an individual fragment into the reference tree is conceptually independent from all other placements, the most time-consuming phase of SEPP can be further parallelized at up to one process per fragment in the extreme case. This design naturally capitalizes on high-performance computing (HPC) environments. The implementation of SEPP, in addition to parallelization, also enables checkpointing (continuing a job from a terminated point), which is important for HPC.

**FIG 10:**
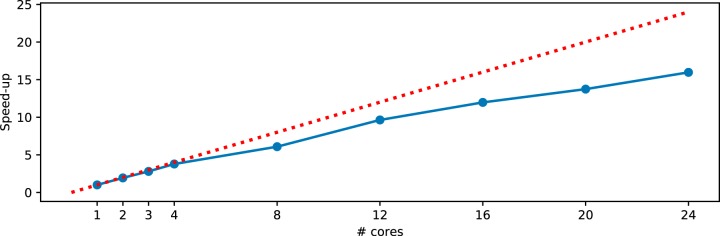
Empirical speedup of SEPP in HPC environments. For a data set with 50,000 fragments, SEPP is used with various numbers of cores on one node of the Comet supercomputing cluster to place fragments into the 99% Greengenes reference tree. The running time starts with 8 h with one thread and continues to decrease with increased numbers of threads. The unit line is shown as a dotted red line.

We provide the “q2-fragment-insertion” BSD-licensed QIIME2 plugin for SEPP for seamless integration into existing analysis pipelines for use either in HPC environments or in environments with stand-alone workstations and laptops. This plugin is Conda installable with a single command. Execution time scales with the number of fragment sequences to be inserted into the reference tree (see Table 1). Memory requirement is dominated by the second phase of SEPP, where all obtained placements are used to insert new tips into the reference tree. Assuming a four-core CPU and 12 GB of RAM such as are typical today for consumer-level computers, SEPP can readily process typical microbiome studies in local environments and can perform large meta-analyses with several hundred thousand sOTUs such as the Earth Microbiome Project (27) in HPC environments in reasonable time (e.g., 4 h 25 min was sufficient time to place ~330-K fragments using 24 cores on the Comet supercomputing cluster). According to Amdahl’s law (28), gains in speedup are limited by the fraction of nonparallelizable code regardless of how many additional CPUs are employed for a constant problem size. Empirical measurements showed that, even with 24 nodes, speedup of SEPP was far from plateauing (see Fig. 10), indicating a high fraction of parallelism and, therefore, a high potential for HPC environments.

**TABLE 1:** Empirical runtime and memory footprint of typical SEPP runs^a^

QIITA ID	No. of sOTUs	No. of samples	sOTU length (nt)	Memory (GB) (max RSS)	Time (h:min)	Wall time (h:min)
1024	21,473	344	150	10.2	06:32	01:54
10315	31,784	199	150	10.2	09:35	02:47
10343	14,245	389	150	10.3	06:10	01:45
10346	108,447	1,292	100	10.4	20:59	06:07
10422	4,702	647	150	10.4	01:37	00:31
2014	23,029	1,017	150	10.2	08:45	02:27
2136	29,702	504	150	10.4	08:48	02:33
550	27,791	1,967	100	10.4	05:49	01:43
850	11,301	528	90	10.2	02:07	00:40
MrOS	4727	599	249	10.3	02:44	00:48

^a^ We ran SEPP on 4/32 cores of an Intel Xeon CPU E5-2640 v3 @ 2.6-GHz server with 265 GB of available RAM. With an ~10-GB memory requirement, SEPP is usable on currently available workstations or laptops. ID, identifier; Time, accumulated “user time"; max RSS, maximum resident set size.

We also integrated SEPP into QIITA, an open-source platform that manages ~2,000 microbial studies with ~500,000 samples. QIITA users interact through a Web browser interface and can process studies in a graphical workflow editor. SEPP is invoked behind the scenes on an HPC cluster and stores fragment placements in a centralized resource, which currently holds ~36 million placements for sOTU fragments of different lengths and regions. This not only reduces the computational burden for new studies, since the number of sOTU fragments without known placements can be significantly dropped by reusing information from the central resource, but might also enable detection of new or poorly resolved taxonomic clades in situations where many fragments are placed into a long branch close to the root of the reference phylogeny. Beyond these plugins, SEPP is also publically available as a stand-alone software program (https://github.com/smirarab/sepp; several reference packages are available at https://github.com/smirarab/sepp-refs/).

## DISCUSSION

As with many technical advances in microbial ecology methods, the sOTU approach provides important advantages (stable, exact-sequence representations of the content of a microbial community obtained from amplicon data) while also introducing disadvantages (in this case, the difficulty of integrating novel sequences into a phylogenetic tree). Here we show that the *de novo* tree approach does not work for integrating the information and that it can lead to apparent incorrect biological conclusions. Similarly, OTU-based approaches lose a substantial proportion of the resolution that is available in the data set (1, 2). However, the SEPP approach provides a scalable method that can integrate information from thousands of studies and, potentially, millions of samples. We recommend SEPP for all sOTU-based studies as representing the best available tradeoff between speed of analysis, maintenance of high-resolution taxonomic information, and the ability to perform accurate phylogenetic diversity analyses that correlate with host phenotype rather than with technical artifacts. Testing whether the same principles apply to other types of environmental samples will be an important focus of future work, although we expect the same concepts to apply across the field of microbial ecology.

## MATERIALS AND METHODS

### V4 fragment generation.

**FIG 11:**
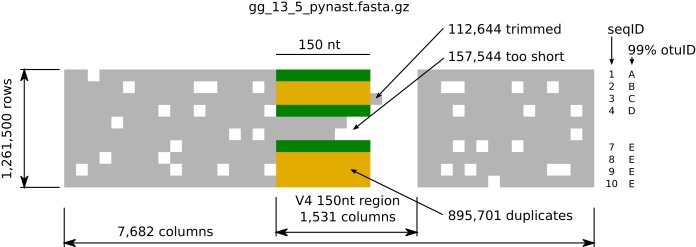
Fragment generation. We degapped a 150-nt V4 region of the PyNAST alignment (from column 2,263 throughout 3,794), trimmed sequences that were too long, and discarded sequences that were too short. Dereplication resulted in 208,255 (green) unique 150-nt V4 fragments.

As described previously (3), we sliced the PyNAST (16) alignment (file gg_13_5_pynast.fasta.gz containing 1,261,500 ribosomal full-length sequences in 7,682 columns) of Greengenes 13.8 to obtain 1,531 *in silico*-determined alignment columns, corresponding to a 150-nt V4 variable region. Closer inspection revealed that 157,544 degapped slices were shorter than the desired 150 nt; thus, those slices were discarded. Additionally, 112,644 degapped slices were too long and were trimmed to the first 150 nt, resulting in 1,103,956 150-nt sequences, 895,701 of which were duplicates. The remaining 208,255 dereplicated sequences constituted our set of V4 fragments (see Fig. 11). Note that the alignment used to generate fragments (the full-length PyNAST alignment) is different from the reference alignment (Greengenes’ small-subunit [SSU] alignment containing all of the sequences but with some positions masked due to high gap frequency) used in SEPP, a fact that can only increase the analytical challenge, due to larger discrepancies between training and testing data.

### Tree constructions.

**FIG 12:**
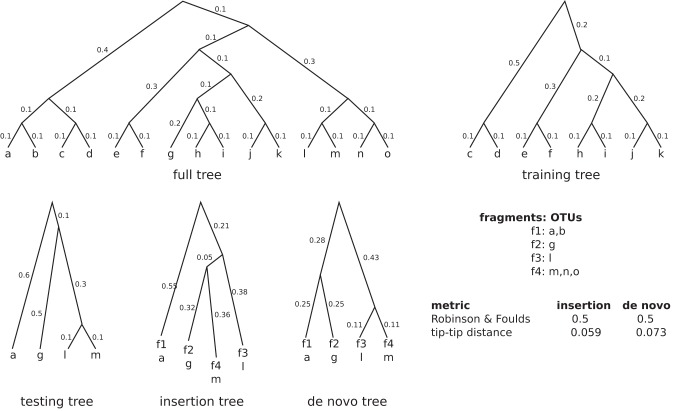
Tree constructions for random reinsertions.

The "full tree" is the 99% OTU Greengenes 13.8 tree with 203,452 tips, with branch lengths recomputed using RAxML (available at https://github.com/smirarab/sepp-refs/releases). The "training tree" is the full tree minus all tips that correspond to fragments that have been chosen for reinsertion. In the example provided in Fig. 12, we chose four fragments (f1, f2, f3, and f4) which are included in the full-length sequences of 7 OTUs, where, e.g., f1 is ambiguously found in OTUs a and b. The "testing tree" has a topology identical to that of the full induced tree down to all but one (query) OTU per fragment to be inserted. Thus, combining the training and testing trees does not necessarily result in the full tree. We give two exemplary trees: first, the insertion tree was a result of using the chosen fragments and inserting them into the training tree and stripping the training OTUs away in a postprocessing step; second, a *de novo* tree was computed in accordance with QIIME2’s recommendation of the four chosen fragments. The lower table shown in Fig. 12 reports actual distances between the testing tree and the insertion or *de novo* tree for the three metrics used.

### SEPP parameters.

SEPP has two main parameters. In the default version used for Greengenes and incorporated into QIIME2, the reference tree is divided into 62 “placement” subsets, each with at most 5,000 tips, and each placement subset is further divided into alignment subsets of at most 1,000 tips to build the HMM examples (292 alignment subsets in total). These choices are driven by computational constraints; increasing the placement subset size (which is in theory desirable) puts a high burden on the memory, and reducing the alignment subset could increase the running time with minor improvements in the accuracy of results (9) (see Fig. S2 in the supplemental material).

10.1128/mSystems.00021-18.1 FIG S1  Perfect matching fragments are precisely inserted below the species level. Data were determined as described for Fig. 5, but here the insertion distance for ambiguous fragments was measured as the smallest distance between the inserted fragment and any of the multiple true originating OTUs. Download FIG S1, EPS file, 0.6 MB. Copyright © 2018 Janssen et al. 2018 Janssen et al. This content is distributed under the terms of the Creative Commons Attribution 4.0 International license.

10.1128/mSystems.00021-18.2 FIG S2  Effects of placement and alignment subset size parameters. We randomly chose 1,000 150-nt V4 fragments to split the Greengenes tree into training and testing trees and ran SEPP with different values for placement and alignment subset sizes to reinsert fragments. We measured the run time in terms of CPU hours, the memory footprint in terms of maximum resident set size (RSS), and the insertion distance as the path length from the inserted position in the tree to the lowest common ancestor (lca) of all true OTU tips. Gray-shaded parameter combinations or missing bars indicate jobs that were terminated due to exceeding the 160-GB memory limit or 12 h of wall time on 20 CPU cores. The pink vertical line indicates species-level diversity in the reference tree. The orange setting (placement subset size of 5,000 and alignment subset size of 1,000) was used throughout this work, while the green setting (10,000/500) yields improved placements. To support computation on local workstations/laptops, we define the orange setting as the default for SEPP. Download FIG S2, EPS file, 0.1 MB. Copyright © 2018 Janssen et al. 2018 Janssen et al. This content is distributed under the terms of the Creative Commons Attribution 4.0 International license.
